# Psychopathological symptoms of patients with heroin addiction entering opioid agonist or therapeutic community treatment

**DOI:** 10.1186/s12991-014-0035-x

**Published:** 2014-11-18

**Authors:** Pier Paolo Pani, Emanuela Trogu, Federica Vigna-Taglianti, Federica Mathis, Roberto Diecidue, Ursula Kirchmayer, Laura Amato, Marina Davoli, Joli Ghibaudi, Antonella Camposeragna, Alessio Saponaro, Fabrizio Faggiano, Angelo Giovanni Icro Maremmani, Icro Maremmani

**Affiliations:** Social and Health Services, Cagliari Health Public Trust (ASL Cagliari), Cagliari, Italy; Department of Psychiatry, Cagliari Health Public Trust (ASL Cagliari), Cagliari, Italy; Piedmont Centre for Drug Addiction Epidemiology, ASLTO3 Grugliasco, Turin, Italy; Department of Clinical and Biological Sciences, University of Turin, Turin, Italy; Department of Epidemiology, Lazio Regional Health Service, Rome, Italy; National Coordination Hospitality Communities (CNCA), Rome, Italy; Regional Epidemiologic Observatory, Emilia Romagna Regional Health Service, Bologna, Italy; Department of Translational Medicine, Avogadro University, Novara, Italy; Department of Neurosciences, Vincent P. Dole Dual Diagnosis Unit, Santa Chiara University Hospital, University of Pisa, Pisa, Italy; Association for the Application of Neuroscientific Knowledge to Social Aims (AU-CNS), Pietrasanta, Lucca, Italy; G. De Lisio Institute of Behavioural Sciences, Pisa, Italy

**Keywords:** Psychopathological symptoms, SCL-90, Residential treatment community, Opioid agonist treatment, Gender differences

## Abstract

**Background:**

The relationship between substance use disorders and psychiatric pathology is still an open question. The main aim of the present study was to verify whether the five psychopathological dimensions identified through the SCL-90 tool in a previous study carried out on patients with heroin addiction entering an outpatient opioid agonist treatment (OAT) were also observable in those entering a residential treatment community (TC). Further aims were to look at differences in the psychopathological profiles of patients entering a TC versus an OAT treatment and at the correlation between gender and the observed psychopathology.

**Methods:**

A confirmatory factor analysis was performed on the results of SCL-90 filled by 1,195 patients with heroin dependence entering TC treatment. It replicates the extraction method previously used on 1,055 OAT patients with heroin addiction by using a principal component factor analysis (PCA). The association between the kind of treatment received (TC or OAT), gender, and the psychopathological dimensions was assessed through logistic regression and general linear model (GLM) analysis.

**Results:**

The PCA carried out on the SCL-90 results of patients entering a TC yielded a five-factor solution, confirming the same dimensions observed in patients entering an OAT: ‘worthlessness and being trapped’, ‘somatization’, ‘sensitivity-psychoticism’, ‘panic anxiety’, and ‘violence-suicide’. The logistic regression analysis showed a statistically significant association between ‘somatization’ and ‘violence-suicide’ severity score and OAT. GLM analysis showed that psychopathological factorial scores for ‘worthlessness-being trapped’, ‘somatic symptoms’, and ‘panic anxiety’ dimensions were more severe in OAT vs TC male patients and in TC vs OAT female ones. ‘Violence suicide’ followed the same severity pattern for males, but did not differ in TC vs OAT females, while ‘sensitivity-psychoticism’ did not differ in OAT vs TC patients. The five dimensions did not differ in OAT males vs females.

**Conclusions:**

Our research appears to confirm the existence of a specific aggregation of psychological/psychiatric features within the category of individuals with heroin addiction. It also shows a correlation between the dominant psychopathological subgroup and the assignment to TC versus OAT. Further research is needed to clarify the differences between the five psychopathological subgroups and their determinants.

## Background

The relationship between substance use and other psychiatric disorders is still an open question. According to current nosographic approaches—especially the one followed by the Diagnostic and Statistical Manual of Mental Disorders (DSM) system, even in its recent fifth version [1]—substance use disorders are defined by the core symptoms of addiction, such as continuation of use despite consequences, reduction of other interests and activities, and craving. Other relevant psychopathological symptoms present in the clinical picture of persons with addictive behaviours are included in separate disorders pertaining to the domain of psychiatric ‘comorbidity’. Several findings challenge this ‘dividing’ approach to unitary clinical presentations: besides the high degree of association between core symptoms of addiction and other psychiatric symptoms [2-4], various neurobiological and clinical considerations highlight the strong link between addiction as such and other forms of psychopathology [5-10]. Accordingly, a revision of the current nosology is needed, moving to approaches based on dimensions of observable behaviour and neurobiological measures [11]. On these bases, an integrated, unified approach, which aims to explain the pathophysiology and phenomenology of addiction, including psychological/psychiatric precursors, acute substance effects, addictive processes, and psychiatric consequences, has been previously proposed [12,13]. Actually, a clinical diagnosis of a psychiatric disorder in the presence of substance addiction is a difficult task. The short- and long-term effects of substances, their withdrawal, and the consequences of addictive processes result in symptoms that cannot be clearly distinguished from those produced by independent psychiatric disorders or psychological conditions preceding substance use. The use of structured and semi-structured interviews has certainly improved the reliability of comorbid psychiatric diagnoses. However, even with the support of these interviews, the reliability of diagnoses remains weak [14-18].

Given this background, and the consequent uncertainty in the correct classification of symptomatology as being intrinsic to the addictive disorder or as due to comorbidity, a low level of inference has been considered in approaching the psychopathology of addiction, while focusing primarily on the symptoms expressed by patients rather than starting from a pre-established syndromic level such as that of the DSM [19-22].

Following this approach, some years ago, our research group applied an exploratory principal component factor analysis (PCA) to symptoms shown by a sample of patients entering an outpatient opioid agonist treatment (OAT) in the city of Pisa [21]. The analysis yielded a three-factor solution identifying 1) a ‘depressive-anxious’ dimension (illness awareness, anxiety state, depressed mood, sleep and eating disturbances), 2) a ‘psychomotor excitement’ dimension (hypomanic/manic or mixed state, aggressiveness and violence, suicidality), 3) and a ‘psychotic state’ dimension, including memory deficits, altered consciousness, delusions, and hallucinations. Adopting a similar approach, Zack et al. applied a PCA to the 90 items listed in the SCL-90 questionnaire administered to a population of 740 outpatients with coexisting substance use (especially alcohol) and psychiatric disorders. This analysis yielded three inter-correlated factors that, taken together, accounted for 38% of the variance found in ratings from the entire sample: the first factor was characterized by anxious and depressive symptoms and identified as ‘general emotional distress’; the second factor reflected a combination of bodily concerns and phobic avoidance and was labelled ‘panic-related symptoms’, and the third factor contained elements of hostile thought and behaviour as well as suspicious beliefs and was identified as ‘hostility and suspiciousness’. In any case, a single higher-order factor was identified that explained 60% of the variance in factor scores. This study also tested the factor structure variations measured by the SCL-90 across gender—a subject on which inconsistencies had been reported in previous studies—so demonstrating a structural similarity between the factors as they appear separately in each gender [20].

In a further investigation carried out by our group, the SCL-90 checklist was used to investigate the psychopathological dimensions of 1,055 patients with heroin addiction who had been admitted to public addiction facilities in Italy and were beginning an OAT. By applying an exploratory PCA to the 90 items in SCL-90, a five-factor solution was identified: the first factor reflected a depressive ‘worthlessness and being trapped’ dimension, the second factor picked out a ‘somatization’ dimension, the third identified a ‘sensitivity-psychoticism’ dimension, the fourth a ‘panic anxiety’ dimension, and the fifth a ‘violence-suicide’ dimension [22]. Overall, the five factors accounted for 37.8% of the variance found between the items.

The main aim of the present study was to verify whether the five psychopathological dimensions identified in the previous study carried out with patients entering an OAT were also observable in subjects with heroin addiction entering a treatment community (TC). Further aims were to look at differences in the psychopathological profile of patients entering a TC versus an OAT treatment and to look at the correlation between gender and the psychopathology expressed through the SCL-90 by the enrolled patients.

Our expectations were that psychopathological subtypes previously observed in opioid dependents would be confirmed in a different sample of opioid dependents, that the severity of patients’ psychopathology would be associated with the treatment modality chosen, and that the psychopathology expressed would be more severe in the female population.

## Methods

### Setting

Patients included in the present study come from two datasets: the first is the Pisa addiction dataset, a database that includes anonymous individual information on patients with heroin addiction receiving OAT at the Italian Addiction Services during the years 1995–2010, information originally collected for clinical or other research purposes. The second is the Evaluation of Therapeutic Community Treatments and Outcomes (VOECT) dataset, which includes individual information on patients with heroin addiction admitted to TC treatment in eight Italian regions in 2008–2009 [23].

### Sample

The OAT sample consisted of 1,055 subjects, with diagnosis of heroin dependence according to DSM-IV criteria; they were evaluated at the time they entered OAT at Italian facilities. The mean age of this sample was 30.6 ± 6.6 years, 83.9% of the subjects were male, 83.1% were single, educational level was high (studies lasting over 8 years) in 10.1% of the sample, 56.6% of subjects were unemployed, and 22.9% were at their first treatment. Heroin history length was 7.3 ± 6.0 years on average.

Patients in the TC cohort (*n* = 1,195) received the diagnosis of heroin dependence according to clinical judgement; they were evaluated at the time they entered the TC. The mean age of this sample was 33.8 ± 7.7 years; 81.4% of the subjects were male, 90.2% were single, educational level was high (studies lasting more then 8 years) in 9.3% of the sample, 72.4% of the subjects were unemployed, and 3.2% were at their first treatment. Heroin history length was 12.2 ± 8.1 years on average.

Considered together, the two samples gave a heterogeneous cohort of 2,250 subjects with a diagnosis of heroin addiction. For the full sample, the mean age was 32.3 ± 7.4 years (range: 15–62). Of the subjects, 1,857 (82.5%) were male, 2,032 (90.3%) had a low educational level (studies lasting 8 years or less), 1,960 (87.1%) were single, 1,516 (67.4%) were unemployed, and 108 (4.8%) were unable to work due to health impairment. The mean duration of addiction was 9.4 ± 7.4 years (min 0.8, max 34.8). A total of 774 (34.4%) had an addiction history lasting less than 5 years, 600 (26.7%) lasting between 5 and 10 years, 358 (15.9%) between 10 and 15 years, 292 (13.0%) between 15 and 20 years, and 225 (10.0%) over 20 years. All these patients were of Italian nationality and were included only once in the sample. In all, 270 (12.0%) were beginning treatment for heroin addiction for the first time.

### Instruments

After its development by Derogatis and colleagues [24], the SCL-90 now includes 90 items, each rated on a 5-point scale of distress. These items can be clustered in nine dimensions: somatization, obsession-compulsion, interpersonal sensitivity, depression, anxiety, anger-hostility, phobic anxiety, paranoid ideation, and psychoticism.

Four global scores can be calculated: 1) Total SCL-90 score (sum of all items); 2) General Symptomatic Index (GSI), the mean score of all recorded items; 3) Positive Symptoms Total (PST), the number of items rated positively; and 4) Positive Symptom Distress Index (PSDI), which is calculated by dividing the sum of all items by the PST score.

Information on other demographic and clinical characteristics of the patients included in the study was collected from clinical records or research questionnaires for the Pisa dataset and from a research questionnaire for the VOECT dataset.

### Data analyses

The two groups of patients (entering OAT or TC services) were compared for socio-demographic and clinical characteristics by means of the chi-square test for categorical variables and of Student’s *t* test for continuous variables. A confirmatory factor analysis was then performed on the SCL-90 results of the 1,195 TC patients, replicating the same extraction method previously used on the 1,055 OAT patients [22]. Put simply, the single factors were extracted by using a PCA (type 2) and then rotating this orthogonally to achieve a simple structure. To limit the number of factors, the criterion used was an eigenvalue >1.5. Items loading with absolute values >0.40 were used to identify the factors. In order to make factor scores comparable, they were standardized into *z* scores. All the subjects were then assigned to a different subtype on the basis of the highest factor score achieved (dominant SCL-90 factor). This procedure gives the opportunity to classify subjects on the basis of the highest symptomatological cluster. At this point, the correlations between the factors extracted in the two populations under study were estimated through Pearson correlation. Lastly, we looked at the inter-rated reliability (Cohen’s kappa) of the factorial analyses, applying the two factor solutions to the TC patients. We considered a Kappa value higher than 0.60 as indicating that the two factor solutions are very similar [25]. Given this similarity, the factor solution identified in the OAT sample was used to compare patients receiving OAT with those receiving therapy in a TC.

The association between dominant psychopathological group and the kind of treatment received (OAT and TC) was assessed through a logistic regression analysis applied to the whole sample of 2,250 subjects resulting from merging the two datasets. In order to take into account differences between the samples, socio-demographic and clinical variables statistically different between TC and OAT patients in the basic analyses were included into the regression model: age, gender, heroin history length, previous treatments, working condition, and welfare benefits.

Finally, the general linear model (GLM) was used to compare psychopathological factorial scores, according to the ‘Group (OAT vs TC) + Gender + Group × Gender’ design, between the two samples of patients, adjusting for age and heroin history length. All analyses were carried out using SPSS v. 4.0 (SPSS, Chicago, IL, USA). Statistical significance was set at the *p* = 0.05 level.

## Results

### Comparison between OAT and TC patients

The TC patients were older (33.87 ± 7.7 years) than the OAT ones (30.56 ± 6.6 years). This difference was statistically significant (*T* = −10.45; *p* = 0.000). Of the sample, 90.2% of the TC patients and 83.1% of the OAT patients were single (chi-square = 23.06; *p* = 0.000); 72.4% and 56.6% were unemployed (chi-square = 35.35; *p* = 0.000); 6.6% and 2.7%, respectively, received welfare benefits (chi-square = 18.23; *p* = 0.000); and 3.2% and 22.9%, respectively, were at their first treatment (chi-square = 196.20; *p* = 0.000). Heroin history length was longer (12.22 ± 8.1 years) in the TC patients than in the OAT ones (7.28 ± 6.0; *T* = −14.28; *p* = 0.000).

Males were 81.4% among the TC patients and 83.9% among the OAT patients. No differences were observed as regards to gender: the female/male ratio was 1:4.3 for patients in the TC sample and 1:5.2 in the OAT patients (*p* = 0.131). Educational level was high (studies lasting over 8 years) in 9.3% of the TC patients and in 10.1% of the OAT ones (*p* = 0.558).

### Confirmatory factor analysis

The PCA applied to the TC sample confirmed a five-factor solution. Altogether, 76 items with a loading >0.40 were retained. ‘Worthlessness’ and ‘being trapped’ were the two leading items in the first depressive dimension; this accounted for 33.3% of the variance. The ‘somatization’ dimension was the second factor, accounting for 3.7% of the variance. The third factor confirmed the ‘sensitivity-psychoticism’ dimension; this accounted for 3.3% of the total variance. Panic symptoms loaded on the fourth factor, the ‘panic-anxiety’ dimension, accounting for 2.3% of the total variance. The last, fifth factor, confirmed a ‘violence suicide’ dimension, which accounted for 2.0% of the total variance. Overall, these five factors accounted for 44.6% of the variance between the recorded items.

On the basis of the highest *z* scores obtained on the five SCL-90 factors (dominant SCL-90 factor), subjects whose dominant factor was ‘worthlessness and being trapped’ included 199 subjects (16.7%); the group with ‘somatization’ as dominant factor comprised 207 subjects (17.3%), the group with ‘sensitivity-psychoticism’ numbered 252 subjects (21.1%), the group with ‘panic-anxiety’ had 350 subjects (29.3%), and the group whose dominant factor was ‘violence-suicide’ was made up of a cluster of 187 subjects (15.5%).

Correlating factorial scores derived from the exploratory and the confirmatory factor analyses, the first factors correlated with a Pearson’s *r* = 0.80, the second with *r* = 0.69, the third with *r* = 0.79, the fourth with *r* = 0.72, and the fifth with *r* = 0.61. All these correlations were statistically significant (*p* < 0.001).

After assigning TC patients to five mutually exclusive groups using exploratory and confirmatory analysis, Cohen’s kappa was 0.60.

As regards the results of the logistic regression analysis carried out on the whole sample obtained by merging the two groups of patients admitted to TC and OAT (Table 1), among the five extracted factors, both the ‘somatic symptoms’ and the ‘violence-suicide’ dominant psychopathological group membership were associated at a statistically significant level with the OAT group membership. Among potential confounding factors, both the female gender and the heroin history length were significantly associated with the TC group, whereas being at first treatment and having a job were associated with the OAT group. Age and presence of welfare benefits did not enter the regression equation.

**Table 1:** **Logistic regression carried out in 2,250 heroin-addicted patients at treatment entry**

	**STEP**	***B***	**Odds ratio**	**Min**	**Max**	***P***
Variables in equation						
Being at first treatment	1	−2.42	0.08	0.05	0.15	0.000
Heroin history length	2	0.008	1.01	1.006	1.009	0.000
Working	3	−0.55	0.57	0.44	0.74	0.000
SCL-90 dominant groups	4					0.000
SCL-90 dominant group (somatic symptom)^a^		−7.48	0.47	0.32	0.69	0.000
SCL-90 dominant group (sensitivity-psychoticism)^a^		−0.19	0.82	0.54	1.23	0.344
SCL-90 dominant group (panic anxiety)^a^		0.16	1.01	0.68	1.51	0.938
SCL-90 dominant group (violence-suicide)^a^		−0.41	0.66	0.43	0.99	0.049
Female gender	5	0.35	1.42	1.03	1.94	0.028
Variables not in equation						
Presence of welfare benefits						
Age						
Statistic: chi-square 321.70, *df* 8, *p* < 001						

It includes SCL-90 factors and other demographic and clinical variables as determinants, and treatment group (TC versus AOT) as a dependent variable.

^a^Considering dominant ‘worthlessness-being trapped’ as reference group.

### Gender and psychopathology

Table 2 shows the differences of SCL-90 indexes between males and females in OAT and TC. Multivariate analysis showed significant differences between the two groups (*F* = 8.66; *df* = 4; *p* = 0.000), the two genders (*F* = 7.31; *df* = 4; *p* = 0.000), and a group by gender interaction (*F* = 8.79; *df* = 4; *p* = 0.000). In particular, total SCL-90 did not differ between the groups; group-gender interaction was statistically significant, with male patients in the TC group showing lower scores and TC females higher ones than the OAT group pairs. The same pattern was observed for GSI. PST was higher in patients in the OAT group than in the TC patients. The same pattern was observed with PSDI. For all indexes, the lowest scores were found among TC males and the highest among TC females. Significant correlations were maintained when the age and duration of dependence were included as covariates (*F* = 2.41; *p* = 001). As the relationship between psychopathological factorial scores and marital status (*F* = 1.09; *p* = 0.35), employment (*F* = 1.19; *p* = 0.31), welfare benefits (*F* = 0.08; *p* = 0.98), and previous treatment (*F* = 1.27; *p* = 0.27) did not reach statistical significance (data not shown), these variables were not included in the model.

**Table 2:** **Differences between OAT and TC patients in SCL-90 indexes by gender**

	**OAT patients**		**TC patients**		**Statistics**
	**Males**	**Females**	**Males**	**Females**	
Total SCL-90	89.67 ± 54.8	89.37 ± 54.5	73.95 ± 55.3	107.02 ± 58.0	Gr: *F* = 0.09; *p* = 0.759
					Ge: *F* = 26.98; *p* = 0.000
					Gr × Ge: *F* = 27.96; *p* = 0.000
General Symptomatic Index	0.99 ± 0.6	0.99 ± 0.6	0.82 ± 0.6	1.18 ± 0.6	Gr: *F* = 0.06; *p* = 0.799
					Ge: *F* = 26.48; *p* = 0.000
					Gr × Ge: *F* = 28.35; *p* = 0.000
Positive symptoms total	48.09 ± 18.1	47.64 ± 18.3	39.92 ± 19.6	50.75 ± 19.0	Gr: *F* = 5.52; *p* = 0.019
					Ge: *F* = 23.12; *p* = 0.000
					Gr × Ge: *F* = 27.36; *p* = 0.000
Positive symptom distress index	1.77 ± 0.7	1.75 ± 0.5	1.69 ± 0.5	1.99 ± 0.5	Gr: *F* = 4.68; *p* = 0.031
					Ge: *F* = 15.71; *p* = 0.000
					Gr × Ge: *F* = 19.32; *p* = 0.000

*Gr* group, *Ge* gender.

Table 3 shows differences between males and females in OAT and TC as regards the five SCL-90 dominant group membership. Multivariate analysis showed significant differences between the two groups (*F* = 16.65; *df* = 5; *p* = 0.000), the two genders (*F* = 76.39; *df* = 5; *p* = 0.000) and group-gender interaction (*F* = 10.09; *df* = 45; *p* = 0.000). In particular, the ‘worthlessness-being trapped’ dimension was more severe in patients in the OAT group than in TC group patients, and in females within the TC group than in males. The same pattern was observed regarding ‘somatic symptoms’, ‘panic anxiety’, and ‘violence-suicide’. ‘Sensitivity-psychoticism’ did not differ between the two groups, and a similar pattern was found between the genders: males of the TC group scored lowest, and females highest. Again, significant correlations were maintained when age and duration of dependence were included as covariates (*F* = 2.87; *p* = 0.000), while the relationship between psychopathological factorial scores and marital status (*F* = 1.04; *p* = 0.39), employment (*F* = 1.41; *p* = 0.21), welfare benefits (*F* = 0.63; *p* = 0.67), and previous treatment (*F* = 0.38; *p* = 0.86) was not statistically significant; therefore, these variables were not included in the model.

**Table 3:** **Differences between OAT and TC patients in SCL-90 indexes by gender**

	**OAT patients**		**TC patients**		**Statistics**
	**Males**	**Females**	**Males**	**Females**	
Worthlessness-being trapped	1.22 ± 0.7	1.20 ± 0.7	1.04 ± 0.7	1.54 ± 0.8	Gr: *F* = 3.76; *p* = 0.052
					Ge: *F* = 28.62; *p* = 0.000
					Gr × Ge: *F* = 33.83; *p* = 0.000
Somatic symptoms	1.28 ± 0.7	1.27 ± 0.8	0.93 ± 0.7	1.35 ± 0.7	Gr: *F* = 10.12; *p* = 0.001
					Ge: *F* = 22.80; *p* = 0.000
					Gr × Ge: *F* = 25.14; *p* = 0.000
Sensitivity-psychoticism	0.82 ± 0.6	0.82 ± 0.6	0.72 ± 0.6	1.01 ± 0.7	Gr: *F* = 1.50; *p* = 0.221
					Ge: *F* = 14.79; *p* = 0.000
					Gr × Ge: *F* = 15.77; *p* = 0.000
Panic-anxiety	0.45 ± 0.5	0.44 ± 0.5	0.37 ± 0.5	0.67 ± 0.6	Gr: *F* = 4.44; *p* = 0.035
					Ge: *F* = 18.30; *p* = 0.000
					Gr × Ge: *F* = 20.70; *p* = 0.000
Violence-suicide	0.94 ± 0.7	1.01 ± 0.7	0.74 ± 0.6	1.00 ± 0.7	Gr: *F* = 6.60; *p* = 0.010
					Ge: *F* = 16.19; *p* = 0.000
					Gr × Ge: *F* = 6.07; *p* = 0.014

*Gr* group, *Ge* gender.

## Discussion

The primary aim of the present study was to verify whether the five psychopathological dimensions identified through the SCL-90 in a previous study carried out on patients entering an outpatient OAT were also observable in patients with heroin addiction entering a residential TC. Additional aims were to look at differences in the psychopathological profile of subjects entering a TC versus an OAT treatment and to look at the correlation between gender and the observed psychopathology.

The factorial analysis applied to the SCL-90 scores of subjects with opioid addiction entering a residential TC yielded the same five-factor solution obtained for subjects with opioid addiction entering an OAT. These five dimensions have been previously discussed in the light of the available literature on the physiopathology and psychopathology of addiction (see Maremmani et al. [22]). Expressed concisely, the first dimension (‘worthlessness and being trapped’) may be explained by the close link between mood disorders and addiction, in terms of neurobiological background, psychological and psychopathological risk factors, and the epidemiology of the two conditions [8,26-32]. The second dimension (‘somatization’) may be accounted for on the grounds of opioid withdrawal symptomatology, which may be correlated with the request for treatment. Withdrawal status may also be involved in the fourth factor (‘panic-anxiety’), due to the overlap between anxiety and withdrawal symptomatology, and shared features in terms of neurobiology and physiopathology [33-35]. The third dimension (‘sensitivity-psychoticism’) may be understood in the light of self-treatment theory, as the consequence of the antipsychotic action of opioids [36-43] or as a consequence of a concomitant abuse of stimulants or cannabis [44-52]. The last dimension (‘violence-suicide’) is marked by impulsiveness, which is a major feature of addictive behaviour, as can be explained by the common neurobiological background, involving the limbic system and prefrontal cortex, and risk factors, such as antisociality and drug-related disinhibition [53-61].

The results of the above confirmatory factor analysis need to be discussed in the light of the heterogeneity of the two samples considered. Actually, the two groups (subjects with heroin addiction entering OAT and those entering residential TC) differ in important factors related to socio-demographic and clinical conditions, treatment setting, and programme characteristics. As regards socio-demographic and clinical characteristics, patients entering OAT or TC did not differ in gender and educational level, but they did differ in age, marital status, employment, welfare benefits, previous treatment, and heroin history length. Patients in the TC group were significantly older, showed a higher percentage of singles, unemployed, and those receiving welfare benefits. They had a statistically significant longer history of heroin use and a greater number of previous treatments than those in the OAT group. As regards settings and the organization of treatment programmes, too, the choices regarding the two groups were clearly divergent: in the first case, there is a highly standardized programme, such as methadone or buprenorphine maintenance, distinguished by its scientifically proven efficacy, governed by guidelines and clear operational procedures [62-64]; in the second, a less standardized and more heterogeneous residential programme, subject to changes and adaptations undertaken to satisfy the needs of a special population (women, adolescents, people affected by psychiatric comorbidity), and financial limitations [65-68].

The TC patients included in our study were drawn from 121 TC setups in eight Italian regions [23]. These communities differ in the variety of the services offered (pharmacological, including opioid agonist treatment, psychological, psychiatric, educational, social, rehabilitative, and work training), for the target population (males, mother-and-child, with a double diagnosis, with polydependence), and in the length of the treatment provided (ranging from a few months to a few years) [23].

In spite of such a high level of heterogeneity in treatment settings and programmes and of the existence of such relevant and statistically significant differences in socio-demographic and clinical characteristics, the SCL-90 patterns obtained through PCA analysis are the same for the two groups, OAT and TCs. According to these results, the five psychological/psychiatric dimensions (‘worthlessness-being trapped’, ‘somatic symptoms’, ‘sensitivity-psychoticism’, ‘panic-anxiety’, ‘violence-suicide’) might be considered as characteristics of the psychopathology of subjects with opioid addiction, regardless of demographic and clinical presentations or treatment choice.

As pointed out above, the presence of specific patterns of psychological features or aggregates of psychiatric symptoms in people with addiction may be explained on the basis of the close neurobiological and neuropsychological links between addiction and other psychiatric conditions such as those pertaining to the mood, anxiety, or impulse control domains [12,13,31,69,70]. Clearly, some of the symptoms involved in the five identified dominant psychopathological groups may depend on the presence of a known comorbid psychiatric disorder or on the presence of a subthreshold mood, anxiety, and/or impulsive/dyscontrol spectrum manifestation. On the other hand, some symptoms may reflect the severity of opioid addiction, as well as the associated use or abuse of, or dependence on other drugs (cocaine, alcohol, or other stimulants and depressants), or a specific pattern of polyabuse. Currently, the longitudinal view of the psychopathology of addictive disorders should take into account a variety of components, such as pre-existing psychological/psychiatric conditions, substance effects/withdrawal symptoms, addictive processes (with psychiatric manifestations related to craving and dyscontrol), and including psychiatric consequences of the interaction between pre-existing conditions and addictive processes [13]. The interaction between all these psychiatric determinants helps to explain why the psychological/psychiatric presentations of people with addiction may diverge from the classical psychiatric nosography and be expressed, rather, through clinical pictures that may resemble, but not be exactly identifiable as, specifically known psychiatric disorders. Due to the deep interaction between different psychiatric determinants, it may be almost impossible to disentangle what pertains to addiction from what pertains to other independent psychopathological features. On these bases, even the ‘dual diagnosis’ construct itself has been questioned, and the need to deepen the investigation to identify the psychic structure of addiction is an issue that has been raised as an immediate concern [13].

When looking at the association between the five psychological/psychiatric dominant groups and the allocation to TC or OAT treatment—taking into account age, gender, heroin history length, previous treatments, working condition, and welfare benefits as potential confounders—‘somatic symptoms’ and ‘violence-suicide’ dominant dimensions are associated with OAT. This could be due to the fact that the somatic dimension, which may be closely correlated with the withdrawal condition, as well as violence and suicide features, may be better handled in an OAT, where the medical approach is stronger. Regarding the other confounding factors considered, it is plausible that patients with a short history of heroin and/or at their first treatment, as well as those who continue to work, may prefer a less demanding treatment such as OAT, which is likely to have a lower impact on their everyday life.

As regards the correlation between gender and psychopathological conditions as caught by SCL-90, while it does not seem to show any impact on patients requiring an OAT, it has an impact on patients undertaking TC: among these, males express a lower and females a higher level of psychopathology than patients entering an OAT. TCs appear to be more easily entered by females with more intense psychiatric symptoms. Differences in psychopathology between females and males with opioid addiction have been previously documented. Previous studies showed that females experience more serious psychological consequences from drug dependence, as well as a greater prevalence and severity of psychopathology than males [71-75]. This higher psychiatric severity is also associated with a higher level of dysfunction in the other areas of life (medical, drug-related, employment, and family/social) [71-73]. The preference of females for the TC treatment might be explained by the potential advantages offered by residential programmes in terms of the alleviation of psychiatric symptoms and the management of multiple needs. This is even more evident nowadays than in the past, since Italian TCs now offer a large variety of treatments, including methadone and buprenorphine treatment, as well as educational, psychological, psychiatric, and rehabilitative interventions.

### Limitations

The psychological/psychiatric profiles of the subjects involved in both the samples were based on self-assessment through SCL-90, an instrument that is easy to administer, requiring few instructions and only a short time for completion. As the retrieved symptoms rely on subjective perceptions, they may differ from those perceived by an expert interviewer. It should be noted that since the patients were enrolled in many different care units, an apparently ‘objective’ evaluation would have not been possible: interviews would have been performed by different interviewers, so involving a high risk of inter-observer differences. We therefore preferred a patient-related self-assessment that allowed the investigation of symptoms in a dimensional perspective, while giving the opportunity to record the experience of the patient without the mediation of the examiner, rather than a non-uniform interviewer-related objective rating. On the other hand, it has to be acknowledged that this kind of patient may have a low level of insight, possibly causing his/her voluntary or involuntary hiding of some symptoms. As a result, caution is needed in interpreting these results, since they may be affected by the lack of any observer-related ‘objective’ evaluation. The use of other instruments to check the tendency of patients to lie, their understanding of the questions that are put to them, and their motivation to participate in the procedure would certainly substantially improve the validity of our work.

Moreover, both samples of patients involved in our research are lacking in any formal psychiatric diagnosis. It must be pointed out that in Italy, psychiatric diagnosis is often formulated late in the course of the treatment received in addiction facilities or local addiction treatment units. In this connection, we have to point out that our research moves from the weakness of categorical psychiatric nosography and from the tendency to attribute symptoms expressed by addicted patients to pre-established psychiatric categories, such as forms of comorbidity. SCL-90 does not allow discrimination in terms of the impact of psychiatric problems: it is likely that a formal and objective psychiatric diagnosis would have made it possible to distinguish between people who do and do not have significant psychiatric conditions and to investigate the relationship between SCL-90-based psychopathological membership and psychiatric diagnosis. As a result, we are not yet in a position to know whether or how strongly the identified profiles are correlated with specific psychiatric diagnostic criteria.

A further limitation is that the SCL-90 questionnaire was administered only at entry into treatment, so results can only be considered representative of subjects with heroin addiction at that particular moment. Some symptoms may vary at different stages of the disease, whereas some may favour or limit certain treatment choices, so that they may prove to be under- or overweighted in our sample.

Moreover, the lack of information on other potential confounders of the association of opioid addiction with the five identified psychological/psychiatric dimensions should be taken into account: we cannot exclude the possibility that the identified profiles were influenced by the presence of opioid withdrawal, by the abuse of, or dependence on, other drugs, or by additional psychiatric disorders. In addition, we do not know whether these five dimensions are stable or whether they may change during the course of addiction.

Furthermore, the OAT sample and the TC cohort show differences in several factors (especially age and length of addiction). In particular, although we adjusted analyses for known confounding factors, the diagnostic procedures followed in the two samples of patients were clearly different: they were DSM-based for patients entering OAT and clinically based for those entering a TC. This may involve a misclassification bias. In this connection, it is important to make the point that given the implications involved in the choice of a residential programme—particularly in terms of limitations on the personal freedom of patients, that often result in dropouts, and higher costs for Italian public services—clinicians are usually required to use a careful approach to the diagnosis of dependence, which may entail the selection of patients who have a severe condition. In any case, the opposite possibility—of the inclusion in the TC sample of patients who did not reflect the full DSM diagnostic criteria—cannot be excluded, either. Therefore, a possible under- or overestimation of the true magnitude of the measures of association used in our study should be taken into consideration.

## Conclusions

By looking at the entire group of answers given by patients with opioid addiction to the SCL-90 questionnaire at their entry into TC treatment, we obtained the same five psychopathological dimensions previously seen in subjects with opioid addiction entering an OAT. While these dimensions may be the expression of the links existing at the aetiological and physiopathological levels between addiction and other mental disorders, at this stage of the investigation, it is too early to draw any firm conclusion on the specific nature or the psychological/psychiatric determinants of the five identified dimensions. Further research is needed to confirm our results, to clarify the differences between the five psychopathological subgroups and their determinants, and to predict symptoms that can benefit from anticraving treatment or that need to be targeted separately.
